# Triangle pore arrays fabricated on Si (111) substrate by sphere lithography combined with metal-assisted chemical etching and anisotropic chemical etching

**DOI:** 10.1186/1556-276X-7-406

**Published:** 2012-07-19

**Authors:** Hidetaka Asoh, Kosuke Fujihara, Sachiko Ono

**Affiliations:** 1 Department of Applied Chemistry, Kogakuin University, 2665-1 Nakano, Hachioji, Tokyo, 192-0015, Japan

**Keywords:** Silicon, Metal-assisted chemical etching, Colloidal crystal templating, Sphere lithography, Anisotropic chemical etching, Triangle pore arrays

## Abstract

The morphological change of silicon macropore arrays formed by metal-assisted chemical etching using shape-controlled Au thin film arrays was investigated during anisotropic chemical etching in tetramethylammonium hydroxide (TMAH) aqueous solution. After the deposition of Au as the etching catalyst on (111) silicon through a honeycomb mask prepared by sphere lithography, the specimens were etched in a mixed solution of HF and H_2_O_2_ at room temperature, resulting in the formation of ordered macropores in silicon along the [111] direction, which is not achievable by conventional chemical etching without a catalyst. In the anisotropic etching in TMAH, the macropores changed from being circular to being hexagonal and finally to being triangular, owing to the difference in etching rate between the crystal planes.

## Background

A number of promising approaches to nanofabrication, which have potential application to high-throughput and low-cost production, are applied to generate patterns on various substrates [1]. The two-dimensional (2D) nano-/micropatterning of solid substrates using self-assembled colloidal particles as a mask, which is often referred to as colloidal lithography or nanosphere lithography, has also attracted considerable attention as a key fabrication method owing to its relative simplicity and low cost. We previously reported the fabrication of ordered silicon microstructures such as silicon convex arrays and silicon nanopore patterns with a regular periodicity on the order of micrometers by combining colloidal crystal templating and site-selective metal-assisted chemical etching using patterned noble-metal particles as catalysts [2]. Because the metal-assisted chemical etching proposed by Li and Bohn in 2000 [3] is a very simple and efficient process that uses no external bias, the number of studies of 2D or three-dimensional (3D) patterning based on metal-assisted chemical etching using a shape-controlled metal catalyst is increasing yearly [4-9]. If a metal catalytic layer with an ordered pore arrangement is applied, the silicon substrate is etched into an array of silicon nanowires [5,8]. Using nanosphere-lithography-based metal-assisted chemical etching, it has been demonstrated by Huang et al. that silicon nanowires with an aspect ratio larger than 30 could be obtained [5]. Regarding 3D patterning, Hildreth et al. demonstrated that the fabrication of 3D silicon nanostructures such as sloping channels, cycloids, and spirals could be achieved by metal-assisted chemical etching using various shape-controlled catalysts (e.g., nanorods, nanodonuts, nanodiscs, nanolines, squares, grids, and star-shaped catalysts) [7]. Using 2D catalyst templates with multiple thicknesses, the fabrication of more complex 3D nanostructures was also achieved by a folding process that combines rotational and translational motion [9].

On the other hand, we have fabricated an ordered arrangement of macropores with diameters above 1 μm in silicon using a circular noble-metal thin film as the catalyst [10,11]. However, the polystyrene honeycomb mask used for metal deposition, which was prepared using binary colloidal crystals composed of large silica spheres and small polystyrene spheres [10], had a relatively coarse framework, resulting in imperfect templating into the silicon substrate. Therefore, to fabricate more precise macroporous silicon using a noble-metal thin film as the catalyst, it is essential to prepare the metal deposition mask with a dense framework and periodic openings. In this work, we used metal-assisted chemical etching through a dense photoresist mask with a 2D hexagonal array of openings, which was prepared by sphere photolithography, to fabricate high-quality macroporous silicon. In addition, we aimed to fabricate triangle pore arrays on a Si (111) substrate using only a circular catalyst by controlling the morphology of the pore shape of etched silicon during anisotropic chemical etching.

## Methods

**Figure 1:**
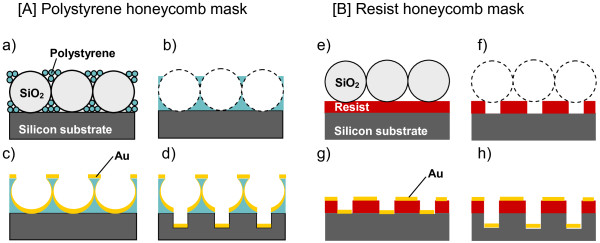
**Fabrication process schematic: ordered silicon macropore arrays using (A) polystyrene and (B) photoresist honeycomb masks.** (**a**) Formation of binary colloidal crystals composed of SiO_2_ and polystyrene spheres on silicon substrate, (**b**) removal of SiO_2_ spheres after heating, (**c**) formation of metal catalyst layer, (**d**) chemical etching of silicon, (**e**) formation of a monolayer of SiO_2_ spheres on the photoresist layer formed on silicon substrate, (**f**) development of resist sites exposed through SiO_2_ spheres, (**g**) formation of metal catalyst layer, and (**h**) chemical etching of silicon.

The principle of the fabrication of silicon macropore arrays is schematically shown in Figure 1. A *p*-type silicon wafer (3 to 5 Ω cm, (111) crystal orientation) was mainly used as the substrate. To compare the morphology of etched silicon, a *p*-type (100) silicon wafer (1 to 20 Ω cm) was also used. In this work, two different types of mask, i.e., (A) a polystyrene honeycomb mask and (B) a resist honeycomb mask, with periodic circular opening arrays, were prepared by colloidal crystal templating. The polystyrene honeycomb mask was prepared by removing the silica spheres used as a template in HF after the formation of binary colloidal crystals composed of large silica spheres and small polystyrene spheres. On the other hand, the resist mask was formed by sphere photolithography using silica spheres as the condenser lens. Concerning the detailed preparation conditions for the polystyrene honeycomb mask [10] and photoresist honeycomb mask [12,13] with honeycomb-like openings, see our previous paper [14].

Au thin films were deposited on silicon substrates by ion sputtering (E-1010, Hitachi High-Tech, Minato-ku, Tokyo, Japan) through a honeycomb mask with a 3-μm periodicity. The sputtering was carried out for 1 min at a discharge current of 15 mA in vacuum with a pressure below 10 Pa. The deposition rate of Au was 10 nm min^−1^. The deposited Au layer was estimated to be a 10-nm-thick continuous circular thin film composed of clusters of Au nanoparticles. After sputtering, the specimens with locally deposited Au films were etched for 1 min in a mixed solution of 15 mol dm^−3^ HF and 1 mol dm^−3^ H_2_O_2_ at room temperature under ambient light. This etchant composition corresponds to *ρ* = [HF]/([HF] + [H_2_O_2_) = 93.8 using the unified notation proposed by Chartier et al. [15]. According to their report, when etching is carried out using a high proportion of HF, i.e., *ρ* > 70%, straight or curved cylindrical pores are formed with a diameter matching the size of the metal nanoparticles embedded at their bottom (in their case, Ag nanoparticles).

To modulate the morphology of the silicon macropores, pre-etched silicon specimens were immersed for a period of either 10, 20, 30, or 40 min in 25% tetramethylammonium hydroxide (TMAH) aqueous solution. After removing the mask by immersing the specimens in toluene for the polystyrene mask and in acetone for the photoresist mask, the morphology of the obtained microstructured silicon was evaluated by scanning electron microscopy (SEM) (JEOL 6701 F, JEOL Ltd., Akishima, Tokyo, Japan).

## Results and discussion

### Two types of mask with periodic circular opening arrays

**Figure 2:**
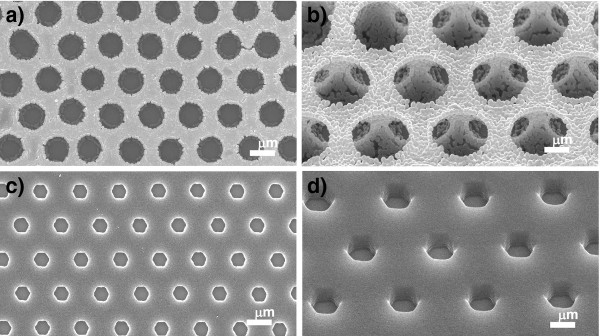
**SEM images of polystyrene honeycomb mask and photoresist honeycomb mask.** (**a, b**) Polystyrene honeycomb mask after removing silica spheres by immersion in 10 wt.% HF for 10 min and (**c, d**) photoresist honeycomb mask formed by sphere lithography. **(a, c)** Low-magnification and **(b, d)** high-magnification images taken at an angle of 45° to the surface.

Figure 2a shows a SEM image of the inverse opal structure derived from using large silica spheres (3 μm in diameter) as the template with small polystyrene spheres (200 nm in diameter). The openings of the honeycomb-like inverse opal structure were arranged hexagonally over the entire area of the specimen. It can be clearly seen in the tilted-view SEM image of Figure 2b that the framework, i.e., the walls of the inverse opal structures, was composed of an aggregation of small polystyrene spheres. In this case, the connections among neighboring polystyrene nanospheres are thought to be produced by heat treatment at 100°C [10]. The center-to-center distance between the openings of the polystyrene honeycomb mask, which was basically determined by the diameter of the large silica spheres, was approximately 3 μm. The diameter of the openings at the bottom part in the polystyrene honeycomb mask, which determines the dimensions of the resultant pattern in secondary fabrication, was approximately 1.5 μm. However, many cracks and voids were observed in the framework of the polystyrene honeycomb mask, especially at the bottom part, as shown in Figure 2b.

On the other hand, the photoresist honeycomb mask, which was prepared by sphere photolithography, had flat and dense walls, as shown in Figure 2c,d. The openings in the resist layer were arranged hexagonally, corresponding to the 2D hexagonal array of silica spheres used as the initial mask to provide information on reactive sites during exposure [13]. The diameter of the openings and the thickness of the resist layer were approximately 1 μm and 850 nm, respectively. The hexagonal contour shape was attributed to interference among adjacent spheres during exposure.

### Formation of macroporous silicon using metal-assisted chemical etching

**Figure 3:**
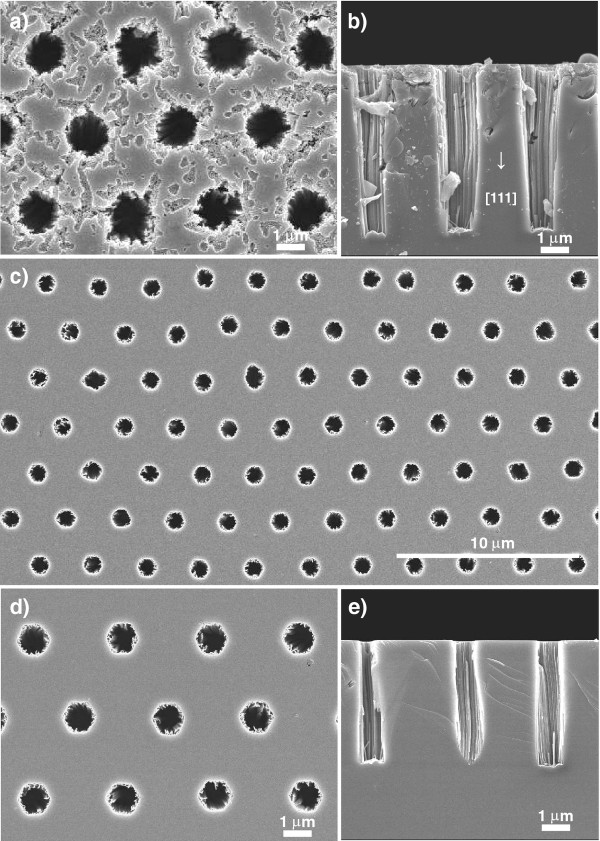
**SEM images of macroporous silicon formed by metal-assisted chemical etching.** SEM images of Au-coated (111) silicon after chemical etching in 15 mol dm^−3^ HF/1 mol dm^−3^ H_2_O_2_ for 1 min using (**a, b**) polystyrene honeycomb mask and (**c** to **e**) photoresist honeycomb mask. The periodicity of the openings of the mask was 3 μm in both cases. **(b, e)** Cross-sectional images of macroporous silicon shown in **(a)** and **(d)**.

The two different types of mask prepared were applied as the metal deposition mask. When ion sputtering was conducted through the mask, isolated circular Au thin films were deposited. The maximum thickness of the Au layer was estimated to be 10 nm [11]. In fact, when the polystyrene honeycomb mask was used as the metal deposition mask, isolated circular Au thin films with a diameter of approximately 1.5 μm were deposited on a silicon substrate. After the deposition of the Au thin films on the Si substrate, the specimens were immersed in a mixed solution of HF and H_2_O_2_ to etch the Si substrate by metal-assisted chemical etching. During chemical etching, the Au-coated Si parts surrounded by the walls of the mask gradually sagged, resulting in the formation of macropores in silicon. After chemical etching for 1 min, small pores and irregular concave features were generated in the surrounding of the main large pores, as shown in Figure 3a. This result implies that small Au particles were deposited on a silicon substrate through defects (e.g., cracks and voids) of the polystyrene mask. To obtain a cross-sectional image by SEM observation, a fracture surface was prepared by mechanical cleavage. From the cross section shown in Figure 3b, it was confirmed that the growth of the pores proceeded in the [111] crystallographic direction. A number of channels on the sidewalls of pores are thought to be reflected in the traces of Au nanoparticles divided from the circular thin film. The depth of the pores reached 6 μm after metal-assisted chemical etching for 1 min. In other words, the etching rate of 6 μm min^−1^ was obtained at room temperature using a metal catalyst. If a bare (111) silicon wafer is immersed in TMAH, which is a popular etchant for the anisotropic etching of silicon, etching at the (111) surface hardly proceeds [16]. In brief, chemical etching in alkaline solution alone cannot form straight pores in the [111] direction. Of course, HF solution is not appropriate for the fabrication of a deep porous structure without external bias. On the other hand, it is notable that etching occurs along the [111] direction if metal catalysts are applied.

Using a resist mask, an ordered array of macropores with a uniform diameter of approximately 1 μm was formed, as shown in the low-magnification view in Figure 3c. The basic chemical etching behaviors were almost the same regardless of the morphology of the mask. The diameter of the pores was almost in agreement with that of the bottom part of the openings in the resist mask. The shape of the pores was sharply defined. Obviously, chemical etching proceeded only in the Au-coated main area on the silicon surface. From the top view shown in Figure 3d, it was confirmed that there were few clear defects at the interspace among the three main pores on the silicon surface, unlike the surface shown in Figure 3a. These results indicate that the adhesion and coverage between the upper mask and the silicon substrate were markedly improved. The usability of the resist mask was also demonstrated for anodic etching in our previous studies [13,17].

Figure 3e shows a cross-sectional image of the macropores. This image indicates that the pores were straight and that the pore depth was approximately 4 μm for metal-assisted chemical etching of 1 min. The etching rate of 4 μm min^−1^ was lower than that described in Figure 3b. The differences in etching rate are considered to be dependent on the differences in size and/or thickness of the catalyst layer caused by sputtering through the two different types of mask.

**Figure 4:**
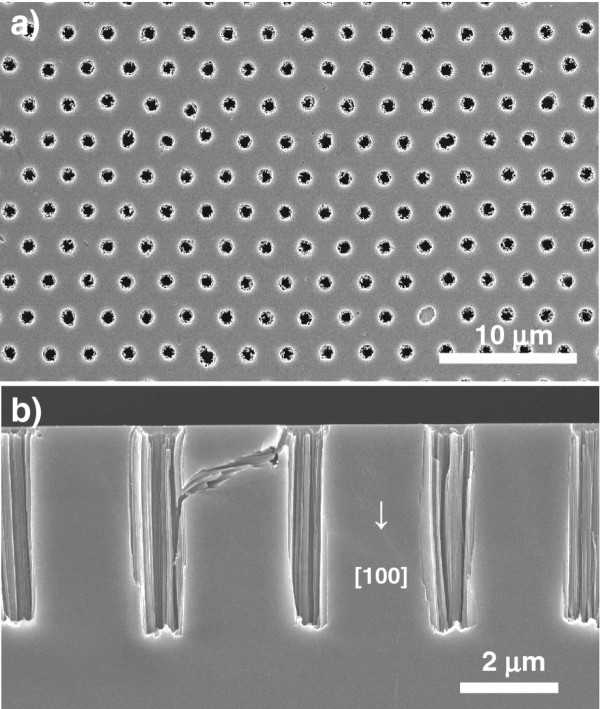
**SEM images of macropore arrays formed in (100) silicon.** (**a**) Plan view and (**b**) cross-sectional SEM images of Au-coated (100) silicon after chemical etching in 15 mol dm^−3^ HF/1 mol dm^−3^ H_2_O_2_ for 1 min using photoresist honeycomb mask. The periodicity of the openings of the mask was 3 μm.

Even when metal-assisted chemical etching was conducted under the same conditions for a *p*-type (100) silicon wafer (1 to 20 Ω cm), similar straight macropore arrays with a 4-μm depth were formed, as shown in Figure 4. In this case, however, the growth of the pores proceeded in the [100] crystallographic direction, i.e., not the [111] direction; that is, the metal-assisted chemical etching proceeded perpendicular to the substrate surface in both cases, independently of the crystal orientation, unlike in conventional chemical etching. For an overview of the effects of the intrinsic properties of the silicon substrate on the etching direction, see the review paper by Huang et al. [18].

### Morphological change of pore shape of etched silicon during post-catalytic etching

**Figure 5:**
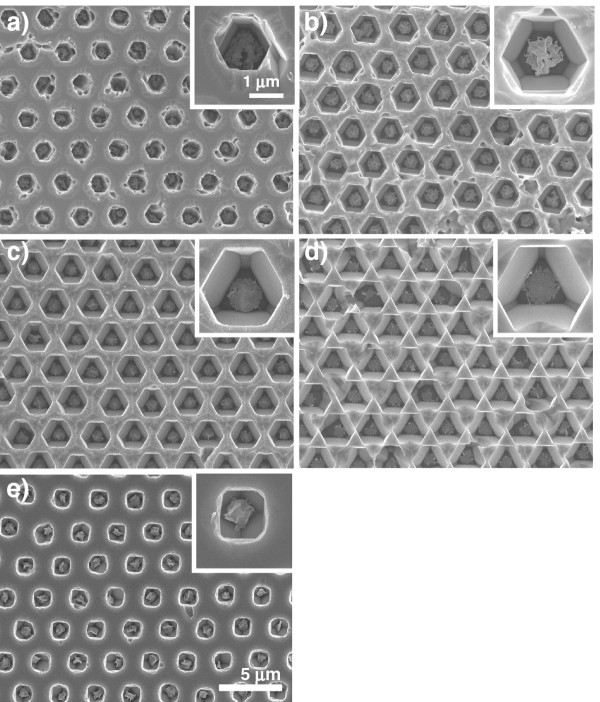
**SEM images of macroporous silicon etched in TMAH at various times.** SEM images of macroporous silicon after chemical etching in 25 wt.% TMAH for (**a**) 10, (**b**) 20, (**c**) 30, and (**d**) 40 min. The inset shows a high-magnification image of each specimen. The Au-assisted chemical etching of (111) silicon was conducted in 15 mol dm^−3^ HF/1 mol dm^−3^ H_2_O_2_ for 1 min through a photoresist honeycomb mask. (**e**) SEM image of Au-coated (100) silicon after metal-assisted chemical etching and subsequent chemical etching in 25 wt.% TMAH for 20 min.

To develop new applications of microstructured silicon, post-catalytic etching in TMAH was attempted to precisely control the pore shape. Here, metal-assisted chemical etching was conducted for (111) silicon for 1 min as pre-etching in advance through a resist honeycomb mask, yielding the straight macropore arrays shown in Figure 3d,e. Figure 5 shows the surface of etched silicon after chemical etching in 25 wt.% TMAH, i.e., so-called anisotropic etching. The etching time was increased from 10 to 40 min to investigate the morphological change of the resultant patterns. After chemical etching for 20 min, the outermost shape of pores changed from a circle to a hexagon. With further chemical etching, the outermost hexagonal opening expanded gradually, and three adjacent pores were placed in contact with each other at a time of 40 min, as shown in Figure 5d. On the other hand, the shape of the bottom part of the macropores changed from a circle to a hexagon and finally to a triangle during anisotropic chemical etching. The Au catalyst remained at the bottom of each pore. This anisotropic aspect of macropores was caused by the difference in etching rate among crystal planes; the etching rate increased in the order of (111) < (100) < (110) planes in TMAH.

**Figure 6:**
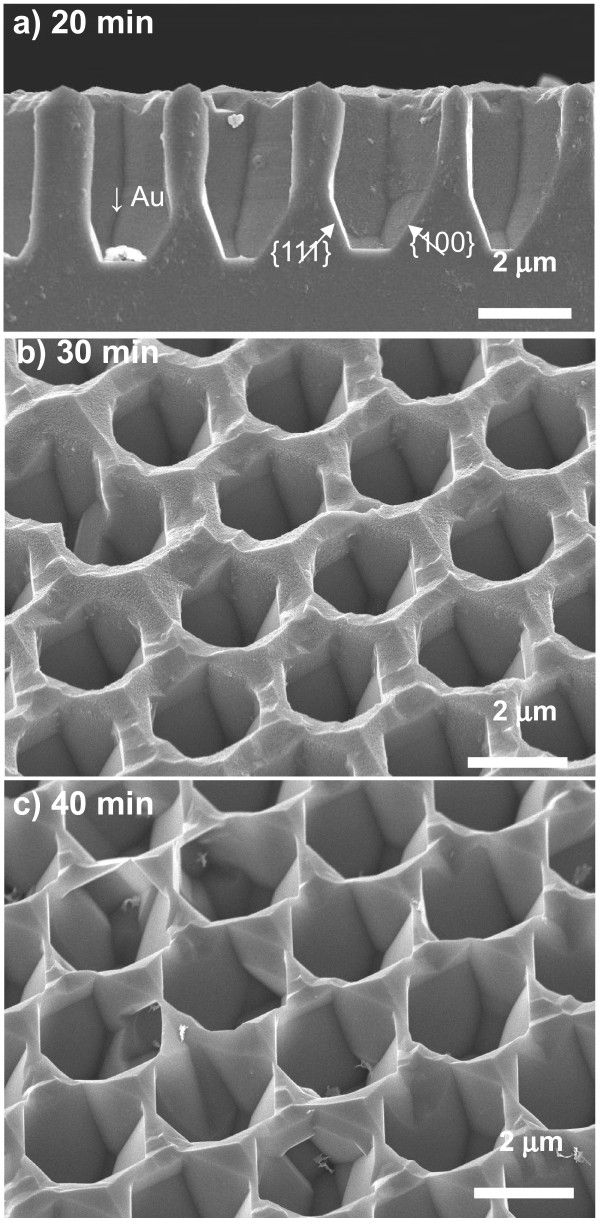
**Morphology of the porous structure in the direction of pore depth.** (**a**) Cross-sectional SEM image of macroporous silicon after chemical etching in 25 wt.% TMAH for 20 min. (**b, c**) Tilted images taken at an angle of 45° to the surface of macroporous silicon after chemical etching in 25 wt.% TMAH for **(b)** 30 and **(c)** 40 min. The conditions for Au-assisted chemical etching of (111) silicon were the same as those in Figure 5.

From the cross-sectional SEM image of macropore arrays in silicon after chemical etching for 20 min in TMAH shown in Figure 6a, the morphology of the porous structure in the direction of pore depth could be observed. The pore depth decreased from approximately 4 to 3.6 μm with chemical etching for 20 min. This result indicates that the top surface was dissolved. In the upper part, the vertical wall is thought to be composed of {110} planes, i.e., the outermost hexagonal pore shape shown in Figure 5b corresponds to {110} planes. On the other hand, at the bottom part, two crystal planes with different angles were observed. Considering the angle between the inclined plane and the basal plane, the two planes are designated as {111} and {100}, respectively. After chemical etching for 30 and 40 min, the {110} and {100} planes disappeared gradually with time in the upper part as shown in Figure 6b,c. Eventually, the three sidewalls and bottom surface were found to consist of {111} planes at the bottom part of each pore.

To investigate the effect of the crystal plane on the morphology of macroporous silicon during post-catalytic etching, metal-assisted chemical etching and a subsequent chemical etching in TMAH were conducted for (100) silicon, under the same conditions as those in Figure 5b. Figure 5e shows a SEM image of the top surface of (100) silicon after chemical etching in 25 wt.% TMAH for 20 min. The outermost shape of pores was a square, not a circle, and a hexagon or a triangle as in the case of (111) silicon. Even under the same etching conditions, the diameter of the pores was smaller than that of the macropores shown in Figure 5b. This result indicates that the difference in the crystal plane directly affects not only the morphology of etched silicon, but also the etching rate in the 2D surface.

**Figure 7:**
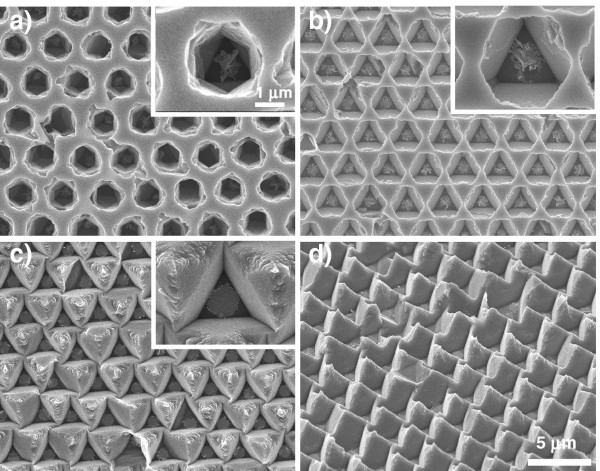
**Effect of TMAH concentration on the morphology of macroporous silicon.** SEM images of macroporous silicon after chemical etching in 1 wt.% TMAH for (**a**) 10, (**b**) 20, and (**c**) 30 min. The inset shows a high-magnification image of each specimen. (**d**) Tilted image taken at an angle of 45° to the surface. The conditions for Au-assisted chemical etching of (111) silicon were the same as those in Figure 5.

Figure 7 shows the surface of etched (111) silicon after anisotropic chemical etching in 1 wt.% TMAH. Even when using a low TMAH concentration of 1 wt.%, a similar morphological change of the etched silicon from a circle to a hexagon and finally to a triangle was observed during anisotropic chemical etching, as in the case using a high TMAH concentration of 25 wt.%. However, the etching rate was approximately twice that in the case of using a high TMAH concentration, as shown in Figure 5. The etched silicon shown in Figure 7b (etching for 20 min in 1 wt.% TMAH) was almost of the same size and shape as that shown in Figure 5d (etching for 40 min in 25 wt.% TMAH). These results indicate that the etching rate increased with decreasing TMAH concentration [16]. After etching for 30 min, the outermost surface was completely dissolved, resulting in the formation of inverted pyramidal pores with a triangular cross section. As the tilted image indicates, the inverted pyramidal pore arrays were reconsidered as ordered silicon pyramid arrays. When the etching time was further increased to 40 min, the pyramid structure was gradually dissolved, and eventually, part of the pyramid arrays disappeared.

## Conclusions

We described the fabrication of ordered macropore arrays in silicon by metal-assisted chemical etching through a dense photoresist mask with a 2D hexagonal array of openings, which was prepared by sphere photolithography. Using the resist honeycomb mask, an ordered array of macropores with a uniform diameter could be fabricated over the entire area of the specimen. The adhesion and coverage of the resist mask to the underlying substrate were significantly higher than those of the polystyrene honeycomb mask prepared using binary colloidal crystals. Furthermore, the shape of the obtained macropores could also be modulated by anisotropic chemical etching in TMAH. Using this technique combined with metal-assisted chemical etching and a subsequent anisotropic chemical etching, the 2D patterning of a silicon surface with well-defined morphology and orientation was achieved. Controlled silicon structures with an ordered periodicity have potential use in not only optoelectronic devices, but also chemical sensors and biofunctional devices that require ordered cavities and high surface-to-volume ratios. We think that this approach provides a beneficial scheme for generating novel patterns for possible applications.

## Abbreviations

SEM, scanning electron microscopy; TMAH, tetramethylammonium hydroxide.

## Competing interests

The authors declare that they have no competing interests.

## Authors’ contributions

HA and SO conceived the idea and designed the experiments. KF carried out all the experiments and data analysis under the instruction of SO. All the authors contributed to the preparation and revision of the manuscript, and read and approved the final manuscript.

## Authors’ information

HA is an associate professor, KF is a graduate student, and SO is a professor at the Department of Applied Chemistry, Kogakuin University.
